# PD-L1 Status in Refractory Lymphomas

**DOI:** 10.1371/journal.pone.0166266

**Published:** 2016-11-18

**Authors:** Semir Vranic, Nilanjan Ghosh, Jeffery Kimbrough, Nurija Bilalovic, Ryan Bender, David Arguello, Yvonne Veloso, Aida Dizdarevic, Zoran Gatalica

**Affiliations:** 1 Department of Pathology, Clinical Center, University of Sarajevo, Sarajevo, Bosnia and Herzegovina; 2 School of Medicine, University of Sarajevo, Sarajevo, Bosnia and Herzegovina; 3 Department of Hematologic Oncology and Blood Disorders, Levine Cancer Institute, Charlotte, North Carolina, United States of America; 4 Caris Life Sciences, Phoenix, Arizona, United States of America; 5 Department of Hematology, Clinical Center, University of Sarajevo, Sarajevo, Bosnia and Herzegovina; Fondazione IRCCS Istituto Nazionale dei Tumori, ITALY

## Abstract

Targeted immunotherapy based on PD-1/PD-L1 suppression has revolutionized the treatment of various solid tumors. A remarkable improvement has also been observed in the treatment of patients with refractory/relapsing classical Hodgkin lymphoma (cHL). We investigated PD-L1 status in a variety of treatment resistant lymphomas. Tumor samples from 78 patients with therapy resistant lymphomas were immunohistochemically (IHC) investigated for the expression of PD-L1 using two antibody clones (SP142 and SP263, Ventana). Thirteen PD-L1+ cases were further analyzed for gene copy number variations (CNV) by NGS and for *PD-L1/JAK2/PD-L2* co-amplification using fluorescent in-situ hybridization assay (FISH). PD-L1 positivity (≥5% positive cancer cells, IHC) was present in 32/77 (42%) and 33/71 cases (46%) using SP142 and SP263 antibodies, respectively. Concordance between the two anti-PD-L1 clones was high with only three (4%) discrepant cases. The strongest and consistent (10/11 cases) expression was observed in cHL and primary mediastinal B-cell lymphomas (3/3). Diffuse large B-cell lymphomas (DLBCL) were frequently positive (13/26) irrespective of subtype. Follicular (1/8), peripheral T-cell (3/11) and mantle cell (1/8) lymphomas were rarely positive, while small lymphocytic lymphoma/CLL and marginal zone lymphomas were consistently negative (3/3). Co-amplification/CNVs of *PD-L1/JAK2/PD-L2* were observed in 3 cases of DLBCL and cHL, respectively. Of note, all three cHL-amplified cases were positive by FISH, but not by NGS. Since only a fraction of the IHC positive lymphoma cases were positive by FISH and NGS assays, other mechanisms are involved in PD-L1 upregulation, especially in DLBCL. FISH assay may be more suitable than NGS assay for determination of *PD-L1* alterations in cHL.

## Introduction

Programmed cell death protein 1 (PD-1, encoded by *PDCD1* gene) and one of its two known ligands, the programmed death ligand-1 (PD-L1, encoded by *CD274* gene) are among the therapeutically most important checkpoint proteins that mediate tumor-induced immune suppression through T-cell downregulation [1]. Their overexpression has been described in various solid tumors with marked clinical therapeutic effects due to the checkpoint blockade [anti-PD1/PD-L1 antibodies] [2], revolutionizing the treatment of solid malignancies, particularly metastatic melanoma, renal cell carcinoma, and non-small cell lung carcinoma (NSCLC).

Patients with relapsed/refractory malignant lymphomas have limited therapeutic modalities and new therapeutic approaches are immensely important [3]. Recent studies revealed the expression of PD-L1 among various B-cell lymphomas [4–6] with the most remarkable therapeutic benefits of PD-1 blockade in patients with Hodgkin lymphoma [3, 7].

PD-L1 status is usually determined by immunohistochemistry [8–10]. Food and Drug Administration (FDA) has recently approved PD-L1 22C3 antibody (DAKO pharmDx) as a companion diagnostics IHC kit for identifying non-small cell lung cancer (NSCLC) patients that are candidates for treatment with pembrolizumab. Several other antibodies (e.g. 28–8 clone from DAKO; SP142 clone and SP263 clone from Ventana) have been developed and used successfully in clinical trials for detection of PD-L1 protein expression in different tumor types (reviewed in [11]). Although PD-L1 overexpression is associated with greater clinical response (particularly to anti-PD1 antibodies) [11], the available clinical data indicate that only 10–30% tumors with PDL1 over expression respond to the PD-1/PD-L1 checkpoint inhibitors [11–14]. The reasons for this discrepancy might be due to different drugs, different antibody clones (validated for specific platforms, e.g. automated Ventana IHC systems or DAKO IHC autostainer), different thresholds, as well as complex pathophysiological mechanisms behind PD-L1 deregulation due to the interactions between cancer and immune cells [10, 15].

Several recent studies investigated the genetic basis of PD-L1 overexpression in tumors. In Hodgkin lymphoma alterations in chromosome 9p24.1 leads to *PD-L1* (CD274) and *PD-L2* (*PDCDLG2*) gene amplification in RS cells [16]. Amplification of *PD-L1* gene has also been described in triple-negative breast carcinomas [17, 18] and NSCLC [19, 20]. Green et al. [16] and Roemer et al. [6] also found *PD-L1* gene alterations in classical Hodgkin lymphoma (cHL) while Georgiou et al. [21] recently demonstrated various cytogenetic alterations of *PD-L1* gene in diffuse large B-cell lymphomas (DLBCL) including gains, amplifications and translocations. Genomic rearrangements of *PD-L1* have also been described in primary mediastinal large B-cell lymphomas [22]. A recent comprehensive survey of Budczies et al. [15] revealed frequent *PD-L1* copy number variations (gains and amplifications [12%], deletions [31%]) across different cancer subtypes with direct impact on its protein and mRNA expression.

In the present study, we explored the expression of PD-L1 in a diverse group of refractory/relapsed lymphomas. We compared the diagnostic utility of two different anti-PD-L1 clones and also explored the genetic basis of PD-L1 overexpression analyzing *PD-L1 (CD274)* gene along with *PD-L2* and *JAK2* genes at 9p24 using in situ hybridization and next-generation sequencing (NGS) assays.

## Materials and Methods

### Samples and patients selection

**Fig 1:**
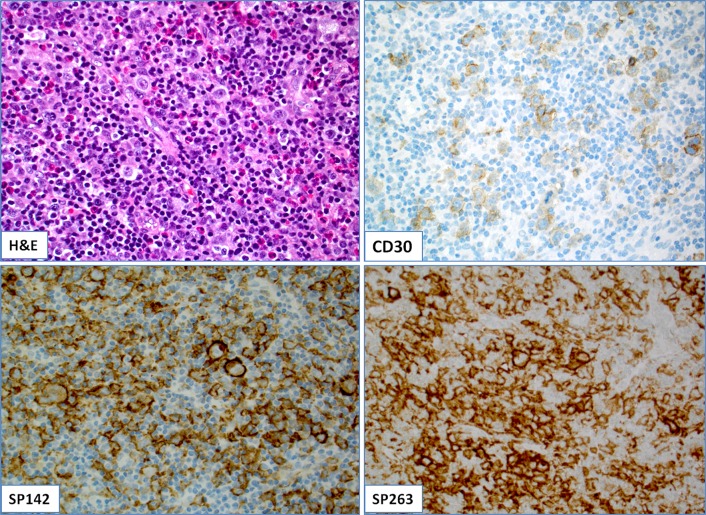
**(A):** A case of cHL with CD30-positive Reed-Sternberg (RS) cells (B); (C-D): The tumor (RS) cells were diffusely and strongly (3+) positive by both SP142 (C) and SP263 anti-PD-L1 clones (D).

The study included 78 patients with refractory and/or resistant lymphomas of both B- and T-cell lineages. All lymphomas were diagnosed by a board-certified hematologist following the most recent lymphoma classification [23]. A comprehensive immunohistochemical examination was performed for all cases for the diagnostic purposes (e.g. CD30 for cHL, Fig 1B). Where appropriate, additional molecular assays (FISH, flow cytometry, PCR) were also performed. Epstein-Barr virus (EBV) status was available for 7 cases of which 5 cases were positive (2 cases of DLBCL of the brain and one case of lymphomatoid granulomatosis, classical Hodgkin lymphoma and peripheral T-cell lymphoma, respectively).

For the study purposes, all samples were re-reviewed by board-certified pathologists (Z.G., N.B., and S.V.) to concur with the diagnosis and appropriate blocks were selected for the study.

The data obtained to conduct the research were obtained from the Caris Life Sciences commercial database, a clinical laboratory system which stores all laboratory results ordered by physicians for standard of care purposes. The analysis was performed in a retrospective fashion utilizing de-identified data created under the Caris honest-broker policy and following consultation with the Caris Life Sciences IRB of record (WIRB). Upon review of the protocol, the Caris Human Subject Compliance Officer confirmed the honest broker policy and verified all data as a de-identified data set. Therefore, the project was deemed exempt from IRB oversight and consent requirements were waived.

### Immunohistochemistry (PD-L1 protein expression)

Formalin-fixed paraffin-embedded tissues from 78 patients with refractory and/or resistant lymphomas of B- and T-cell lineages were investigated for the expression of PD-L1 (Clones: SP142 and SP263, Ventana Medical Systems, Tucson, AZ) using automated immunohistochemical methods at a CLIA/CAP/ISO15189—certified lab (Caris Life Sciences, Phoenix, AZ). Cases were considered positive when ≥5% of the neoplastic cells exhibited membranous positivity with 2+/3+ intensity [24]. PD-L1 expression was also evaluated in adjacent population of reactive/inflammatory cells.

Two pathologists (Z.G. and S.V.) evaluated the IHC results independently; in a case of discrepant data, the cases were reviewed together and consensus was obtained.

### *PD-L1 (CD274)* gene status

Thirteen PD-L1 positive cases by IHC with available tissue were further studied for PD-L1 (*CD274*) gene status using NGS and/or FISH.

### Fluorescent in situ hybridization (FISH)

Nine cases (5 cHL, 3 DLBCL and one case of peripheral T-cell lymphoma NOS) were tested by FISH.

For FISH assay a 586kb probe was designed to cover the *JAK2/PD-L1/PD-L2* gene region at 9p24.1 (chr9:4985240–5571285 (Hg19, Feb.2009)). A second probe was designed to the peri-centromeric region of chromosome 9 (chr9:38079360–38446085 (Hg19, Feb.2009)) as a chromosome copy number control. Both probes were designed to be free of repetitive sequences and synthesized using Agilent’s oligo-based SureFISH technology. The 9p24.1 and peri-centromeric probes were labeled with Texas Red and fluorescein fluorochromes respectively.

### Copy number variations (CNV) by Next-generation sequencing (NGS)

Ten cases (4 cases of cHL, 4 DLBCL, and one case of lymphomatoid granulomatosis and NK/T-cell nasal-type, respectively) were analyzed for copy number variations (CNV) using NGS assay (Illumina NextSeq® System for sequencing). For detection of gene amplification, the average coverage depth of each exon (excluding low mapping quality reads) was calculated. The depth was normalized by dividing with trimmed sample mean (calculated with non-sex chromosome regions for each sample after excluding top and bottom 5% of exons), and transformed to log2 scale. For each region (exon), an average value and standard deviation were pre-computed with data of hundreds of reference samples generated from matching laboratory process [592-gene NGS assay (Agilent SureSelect XT)], after removing outliers. Underperforming regions were excluded to ensure that the reportable regions were created from high quality data. A region was considered amplified if the normalized region depth was greater than or equal to 6 copies above the background ploidy. In order for a gene to be called amplified, a predefined proportion (90%) of the regions within that gene should be called amplified. The final output file included gene-level calls and region-level calls for each case.

Batch sequencing run QC includes Cluster Density (supported number of clusters created per mm2 in appropriate range ~150–300); Reads Passing filter (Number of reads included in the data production) >65%; Q30 score—99.9% of base calls are accurate (%>Q30 Phred Quality score). Also the batch controls (positive Wild Type, Positive sensitivity control and negative control) must pass and produce acceptable and expected results. From a sample to sample passing quality metric, each sample must have >400 average depth of coverage overall or the case is repeated or reported out as indeterminate.

### Statistical methods

The χ2 test was used for comparisons of the variables and groups while the Spearman correlation rank was applied for the correlation between the variables. The differences were considered significant at a p-value <0.05. All data were analyzed using IBM SPSS v.19 (IBM Corp., Armonk, NY, USA).

## Results

### Clinicopathologic characteristics of the cohort

The study included samples from 78 patients (35 female and 43 male patients) diagnosed with refractory and/or relapsed lymphomas of B- and T-cell lineages. The mean patient’s age was 55.6 years (range, 18–86 years). Histotypes included 11 cases of cHL (7 cases of nodular sclerosis, and 2 cases of mixed cellularity and lymphocyte-depleted cHL, respectively), 26 cases of DLBCL [DLBCL of the brain (n = 10); ABC type (n = 6); GCB type (n = 3); DLBCL not specified (n = 7)], 9 cases of mantle cell lymphomas, 8 cases of follicular [FL]; 4 cases of marginal zone lymphoma; three cases of primary mediastinal B-cell lymphoma and small lymphocytic/chronic lymphocytic leukemia [SLL/CLL], respectively; single cases of lymphomatoid granulomatosis [LG], mixed FL/DLBCL, and B-cell lymphoma (not specified). The study also included 11 peripheral T-cell lymphomas [7 cases of PTCL NOS and one case of hepatosplenic T-cell lymphoma, angioimunoblastic T-cell lymphoma, anaplastic large T-cell lymphoma (ALK-negative) and NK/T-cell nasal type lymphoma, respectively].

### PD-L1 expression in lymphomas by immunohistochemistry (IHC)

Results of PD-L1 expression were available for 77 cases while 71 cases had interpretable results for both anti-PD-L1 clones (summarized in Tables 1 and 2 and S1 File).

**Table 1:** Overview of PD-L1 expression by different anti-PD-L1 clones across the different subtypes of lymphomas.

Histotype/PD-L1 status	SP142 clone (n = 77)	SP263 clone (n = 71)
**cHL (n = 11)**	10/11 (91%)	10 (91%)
**DLBCL (n = 26)**	13/26 (50%)	13/23 (57%)**
**FL (n = 8)**	1/8 (12.5%)	1/6 (17%)
**MCL (n = 9)**	0/9 (0%)	1/8 (12.5%)**
**PTCL (n = 11)**	3/11 (28%)	3/11 (28%)
**MZL (n = 3)**	0/3 (0%)	0/3 (0%)
**SLL/CLL (n = 3)**	0/3 (0%)	0/3 (0%)
**PMBCL (n = 3)**	3/3 (100%)	3/3 (100%)
FL/DLBCL (n = 1)*	1/1 (100%)	1/1 (100%)
**LG (n = 1)**	1/1 (100%)	1/1 (100%)
**BCL-NOS (n = 1)**	0/1 (0%)	0/1 (0%)

cHL = Classical Hodgkin lymphoma; DLBCL = Diffuse large B-cell lymphoma; FL = Follicular lymphoma; MCL = Mantle cell lymphoma; PTCL = Peripheral T-cell lymphoma; MZL = Marginal zone lymphoma; SLL/CLL = Small lymphocytic lymphoma/chronic lymphocytic leukemia; PMBCL = Primary mediastinal B-cell lymphoma; LG = Lymphomatoid granulomatosis; BCL NOS = B-cell lymphoma, not specified.

*Case of mixed FL and DLBCL with PD-L1 positivity in DLBLC component. FL component was negative for PD-L1 with both antibodies.

**Indicates discrepant cases (two DLBCLs and one mantle cell lymphoma were positive for PD-L1 using SP263, but not SP142 clone).

**Table 2:** An excellent concordance between SP142 and SP263 antibodies was observed with only three discrepant cases (4%) including two cases of diffuse large B-cell lymphoma and one case of mantle cell lymphoma.

Antibodies	SP263 clone		Total
SP142 clone	Negative	positive	
**Negative**	39	3	42
**Positive**	0	30	30
**Total**	39 (54.2%)	33 (45.8%)	72 (100%)

**Fig 2:**
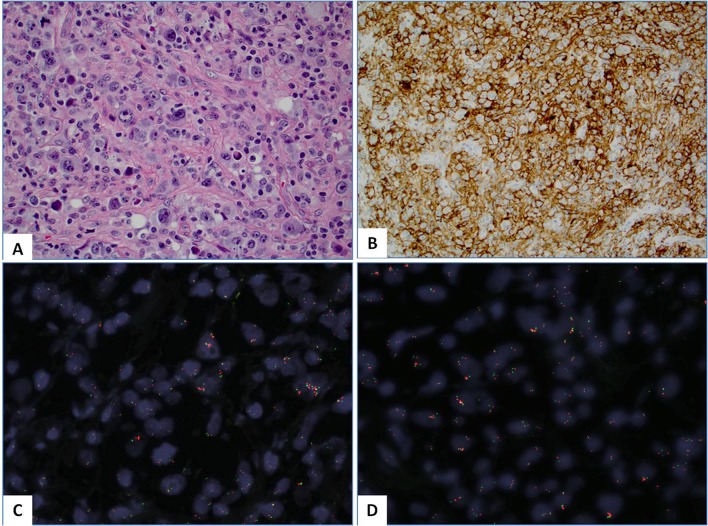
**(A)**: Hematoxylin and Eosin (H&E) slide of a case of lymphocyte depleted variant of classical Hodgkin lymphoma (cHL); **(B)**: The tumor cells were diffusely (100%) and strongly (3+) positive for PD-L1 protein by immunohistochemistry (clone: SP142); **(C-D)**: FISH) assay revealed the *PD-L1* and PD-L2 gene amplification (>6 *PD-L1* copies per tumor cell) (C) and *JAK2* gene (D). Note that red signals designate the probe that covers *PD-L1/PD-L2/JAK2* genes at 9p24.1 while the green signals highlight the peri-centromeric region of the chromosome 9.

Overall PD-L1 positivity (≥5% positive cancer cells with 2+/3+ intensity) was 32/77 (42%) by SP142 and 33/72 (46%) by SP263 antibody (Table 1). The highest PD-L1 positivity was observed in cHL (10/11, 91%; one case of nodular sclerosis cHL was completely negative) (Figs 1C, 1D and 2B) and primary mediastinal B-cell lymphomas (3/3, 100%). Diffuse large B-cell lymphomas (DLBCL) were frequently positive (13/26, 50%) irrespective of subtype. Follicular (1/8), peripheral T-cell (2/11) and mantle cell (1/8) lymphomas were rarely positive, while small lymphocytic lymphoma/CLL and marginal zone lymphomas were consistently negative. A single case of DLBCL arising in FL exhibited positivity exclusively in DLBCL component (~25% by 2–3+ intensity by both antibodies) while FL component was devoid of PD-L1 expression (Table 1).

The mean percentage of PD-L1 positivity in tumor cells was 21% (range, 5–100%). Seven out of 10 positive cHL (70%) had PD-L1 positivity in 100% of the neoplastic cells (Figs 1C, 1D and 2B) as well as one case of primary mediastinal B-cell lymphoma in contrast to only 3 out of 13 positive DLBCL (23%). Three negative cases (one case of DLBCL, MCL and PTCL NOS, respectively) exhibited PD-L1 at 1% of neoplastic cells.

We also evaluated PD-L1 positivity in adjacent inflammatory (reactive) cell population; Fifty-five out of 58 cases (95%) had PD-L1 positivity in reactive/inflammatory cells (ranging from single cells to abundant reactive population as typically seen in cHL; Figs 1C, 1D and 2B); only 3 cases had no visible PD-L1+ reactive cells by both clones.

### Concordance between SP142 and SP263 clones

The mean overall tumor cell positivity for PD-L1 by SP142 clone was 20.46% and 25.45% by SP263 clone. As expected, the percentage of positive tumor cells was significantly higher in cHL (RS cells and variants) in comparison with NHL positive cases (p<0.001, Chi-square test). We found an excellent concordance between SP142 and SP263 clones (Fig 1C and 1D) with only three discrepant cases (4%) including two cases of DLBCL and one case of mantle cell lymphoma (Spearman’s correlation coefficient was 0.919 indicating a high association between the two assays). In the discrepant cases staining for PD-L1 was positive using SP263 clone in 5% of the tumor population with moderate (2+) intensity (Table 2).

### *JAK2/PD-L1/PD-L2* genes’ status by FISH and NGS assays

Results of NGS/FISH assays are summarized in Table 3.

**Table 3:** Summary of the NGS and FISH assays.

Case (lymphoma subtype)	NGS			FISH
	*PD-L1*	*JAK2*	*PD-L2*	
**Case#1 (cHL)**	Not amplified	Not amplified	Not amplified	Amplified*
**Case#2 (cHL)**	Not amplified	Not amplified	Not amplified	Amplified*
**Case#3 (DLBCL)**	Amplified	Not amplified	Amplified	Not available
**Case#4 (NK/T-cell)**	Not amplified	Not amplified	Not amplified	Not available
**Case#5 (LG)**	Not amplified	Not amplified	Not amplified	Not available
**Case#6 (cHL)**	Not amplified	Not amplified	Not amplified	Amplified*
**Case#7 (cHL)**	Not amplified	Not amplified	Not amplified	Not amplified
**Case#8 (DLBCL)**	Not amplified	Not amplified	Not amplified	Not amplified
**Case#9 (DLBCL)**	Amplified	Amplified	Amplified	Amplified*
**Case#10 (cHL)**	Not available	Not available	Not available	Not amplified
**Case#11 (PTCL)**	Not available	Not available	Not available	Not amplified
**Case#12 (DLBCL)**	Not available	Not available	Not available	Amplified
**Case#13 (DLBCL)**	Not amplified	Not amplified	Not amplified	Not available

*indicates discrepancy between the NGS and FISH assays.

NGS = Next-generation sequencing; FISH = Fluorescent in situ hybridization; cHL = Classical Hodgkin lymphoma; DLBCL = Diffuse large B-cell lymphoma; PTCL = Peripheral T-cell lymphoma; NK/nasal type = NK/T-cell nasal type; LG = Lymphomatoid granulomatosis.

Six out of 13 tested cases (46%) were positive by NGS or FISH (three cases of cHL and DLBCL, respectively). Two out of 10 tested cases by NGS exhibited co-amplification at 9p24.1: One case (DLBCL) harbored co-amplification of *PD-L1* and *PD-L2* without *JAK2* gene alterations while another DLBCL case had co-amplification of all three genes. Among the 8 negative cases, four cases were cHL. These cases were further tested by FISH and three of them (75%) exhibited co-amplification of *PD-L1/JAK2/PD-L2* genes (Fig 2C and 2D); two cases exhibited low amplification (~4 copies of *PD-L1* gene) while the third case harbored ≥6 copies of *PD-L1* per tumor cell. One of the positive DLBCL cases was also confirmed by FISH while another, non-amplified DLBCL case by NGS exhibited no 9p24.1 alterations by FISH.

## Discussion

Immune check point blockade with PD-1/PD-L1 has dramatically changed the cancer treatment paradigm with impressive results in several solid tumors as well as in cHL. PD-L1 overexpression has also been described in cancer subtypes beyond those that have been so far considered for immune check point inhibitors [4, 10, 24–26], potentially opening this type of therapy to a larger and more diverse populations of cancer patients. However, predictive value of immunohistochemical biomarkers (thresholds, cellular distribution, methods of analysis) for the PD-1/PD-L1 axis inhibition need still be refined. In some lymphoma subtypes, the identification of PD-L1 positive tumor cells may be challenging due to the abundant reactive/inflammatory cells (e.g. peripheral T-cell lymphomas and cHL). Also, we observed different distribution of PD-L1 expression across the lymphoma subtypes; thus, cHL usually exhibit diffuse and strong PD-L1 positivity on tumor (RS) cells while other lymphoma subtypes rarely exhibit such a pattern of PD-L1 expression.

Patients with relapsed/progressive malignant lymphomas have limited therapeutic modalities and new therapeutic approaches are of crucial importance. Our study revealed over-expression of PD-L1 in cHL, DLBCL (including primary mediastinal B-cell lymphomas), a subset of peripheral T-cell lymphomas and a single case lymphomatoid granulomatosis. These findings are consistent with comprehensive surveys of Menter et al. [clones E1L3N (Cell Signaling, Danvers, MA, USA) and SP142 (Roche/Ventana, Rotkreuz, Switzerland) [5] and Chen et al. (clone 15, #10084-R015, Sino Biological) [4], both of which included a large number of both Hodgkin and non-Hodgkin lymphomas. Another important finding of our study was an excellent concordance between SP142 and SP263 clones using the 5% cut-off as recommended [13, 24]. There are only a few studies that have analyzed the diagnostic utility and comparison of different anti-PD-L1 antibody clones [5, 10, 27–29]. A systematic review of Carbognin et al. [13] confirmed a significant difference in clinical response among solid tumors (melanoma, NSCLC, genitourinary cancers) when a 5% cut-off is used instead of 1% threshold. Hence, development of a standardized threshold for PD-L1 expression in lymphomas requires further investigation.

There is increasing number of studies indicating that alterations at 9p24.1 affect PD-1 ligands (“9p24.1 amplicon”), particularly *PD-L1* gene [6]. This amplicon also contains *JAK2 (Janus kinase 2)* gene involved in JAK2-STAT signaling that further may activate *PD-L1* [16] and *PD-L2* gene, which is another ligand of PD-1. Similar to previous data, we showed that a subset of PD-L1+ lymphomas (both cHL and non-Hodgkin lymphoma) may harbor genetic alterations at 9p24.1 amplicon, which implies that the remaining non-amplified cases may have “adaptive” PD-L1 overexpression without underlying *PD-L1* gene alterations [15]. One case harbored co-amplification of *PD-L1* and *PD-L2* without *JAK2* gene alterations, which is in line with a recent study of Budczies et al. [15]. The authors showed common *PD-L1* copy number variations (gains and amplifications [12%], deletions [31%]) across different cancer subtypes with direct impact on its protein and mRNA expression. In particular, cHL are characterized by consistent *PD-L1/PD-L2* gene alterations [6]. One of the important exploratory findings in our study is diagnostic utility of FISH assay for detection of *PD-L1* gene alterations in cHL. FISH method appears to be a more suitable methodology than NGS for assessing 9p24 status in tumors with a low cancer cell density as it allows for morphologic (visual) identification of single amplified RS cells within heterogeneous (reactive) cell populations in cHL.

We conclude that a substantial proportion of relapsed/refractory B-cell and T-cell non-Hodgkin lymphomas and nearly all classic Hodgkin lymphomas overexpress PD-L1 protein, implying a potential utility of checkpoint blockade therapy in these difficult to treat diseases. Anti-PD-L1 clones SP142 and SP263 exhibit an excellent concordance and both antibodies may be used for IHC detection of PD-L1 in tumors. We also confirm that a subset of refractory, PD-L1 positive lymphomas may harbor genetic alterations of 9p21.4 amplicon affecting *PD-L1*, *PD-L1* and *JAK2* genes. FISH assay may be more suitable than NGS assay for determination of *PD-L1* alterations in cHL.

## Supporting Information

S1 File Lymphoma database with results. (XLSX)

## References

[1] Voena C, Chiarle R. Advances in cancer immunology and cancer immunotherapy. Discov Med. 2016; 21:125–33. 27011048

[2] Hubbard-Lucey VM, Tontonoz MJ. Translating Science into Survival: Report on the Inaugural International Cancer Immunotherapy Conference. Cancer Immunol Res. 2016; 4:3–11. 27119139 10.1158/2326-6066.cir-15-0279

[3] Ansell SM, Lesokhin AM, Borrello I, Halwani A, Scott EC, Gutierrez M, et al PD-1 blockade with nivolumab in relapsed or refractory Hodgkin's lymphoma. N Engl J Med. 2015; 372:311–9. 10.1056/NEJMoa1411087 25482239 PMC4348009

[4] Chen BJ, Rodig SJ, Ouyang J, Sun HH, Roemer MG, Xu ML, et al PD-L1 expression is characteristic of a subset of aggressive B-cell lymphomas and virus-associated malignancies. Clin Cancer Res. 2013; 19:3462–73. 10.1158/1078-0432.CCR-13-0855 23674495 PMC4102335

[5] Menter T, Bodmer-Haecki A, Dirnhofer S, Tzankov A. Evaluation of the diagnostic and prognostic value of PDL1-expression in Hodgkin- and B-cell lymphomas. Hum Pathol. 2016; 54:17–24. 10.1016/j.humpath.2016.03.005 27045512

[6] Roemer MG, Advani RH, Ligon AH, Natkunam Y, Redd RA, Homer H, et al PD-L1 and PD-L2 Genetic Alterations Define Classical Hodgkin Lymphoma and Predict Outcome. J Clin Oncol. 2016; 34:2690–7. 10.1200/JCO.2016.66.4482 27069084 PMC5019753

[7] Moskowitz CH, Ribrag V, Michot JM, Martinelli G, Zinzani PL, Gutierrez M, et al PD-1 blockade with the monoclonal antibody pembrolizumab (MK-3475) in patients with classical Hodgkin lymphoma after brentuximab vedotin failure: preliminary results from a phase Ib study (KEYNOTE-013). Blood. 2014; 124:290.

[8] Taube JM, Klein A, Brahmer JR, Xu H, Pan X, Kim JH, et al Association of PD-1, PD-1 ligands, and other features of the tumor immune microenvironment with response to anti-PD-1 therapy. Clin Cancer Res. 2014; 20:5064–74. 10.1158/1078-0432.CCR-13-3271 24714771 PMC4185001

[9] Patel SP, Kurzrock R. PD-L1 Expression as a Predictive Biomarker in Cancer Immunotherapy. Mol Cancer Ther. 2015; 14:847–56. 10.1158/1535-7163.MCT-14-0983 25695955

[10] Gatalica Z, Snydder C, Maney T, Ghazalpour A, Holterman DA, Xiao N, et al Programmed cell death (PD-1) and its ligand (PD-L1) expression in common cancers and their correlation with molecular cancer type. Cancer Epidemiol Biomarkers Prev. 2014; 23:2965–70. 10.1158/1055-9965.EPI-14-0654 25392179

[11] Kerr KM, Hirsch FR. Programmed Death Ligand-1 Immunohistochemistry: Friend or Foe? Arch Pathol Lab Med. 2016; 140:326–31. 10.5858/arpa.2015-0522-SA 26756647

[12] Gandini S, Massi D, Mandalà M. PD-L1 expression in cancer patients receiving anti PD-1/PD-L1 antibodies: A systematic review and meta-analysis. Crit Rev Oncol Hematol. 2016; 100:88–98. 10.1016/j.critrevonc.2016.02.001 26895815

[13] Carbognin L, Pilotto S, Milella M, Vaccaro V, Brunelli M, Caliò A, et al Differential Activity of Nivolumab, Pembrolizumab and MPDL3280A according to the Tumor Expression of Programmed Death-Ligand-1 (PD-L1): Sensitivity Analysis of Trials in Melanoma, Lung and Genitourinary Cancers. PLoS One. 2015; 10:e0130142 10.1371/journal.pone.0130142 26086854 PMC4472786

[14] Nanda R, Chow LQ, Dees EC, Berger R, Gupta S, Geva R, et al Pembrolizumab in Patients With Advanced Triple-Negative Breast Cancer: Phase Ib KEYNOTE-012 Study. J Clin Oncol. 2016; 34:2460–7. 10.1200/JCO.2015.64.8931 27138582 PMC6816000

[15] Budczies J, Bockmayr M, Denkert C, Klauschen F, Gröschel S, Darb-Esfahani S, et al Pan-cancer analysis of copy number changes in programmed death-ligand 1 (PD-L1, CD274)—associations with gene expression, mutational load and survival. Genes Chromosomes Cancer. 2016; 55:626–39. 10.1002/gcc.22365 27106868

[16] Green MR, Monti S, Rodig SJ, Juszczynski P, Currie T, O'Donnell E, et al Integrative analysis reveals selective 9p24.1 amplification, increased PD-1 ligand expression, and further induction via JAK2 in nodular sclerosing Hodgkin lymphoma and primary mediastinal large B-cell lymphoma. Blood. 2010; 116:3268–77. 10.1182/blood-2010-05-282780 20628145 PMC2995356

[17] Barrett MT, Anderson KS, Lenkiewicz E, Andreozzi M, Cunliffe HE, Klassen CL, et al Genomic amplification of 9p24.1 targeting JAK2, PD-L1, and PD-L2 is enriched in high-risk triple negative breast cancer. Oncotarget. 2015; 6:26483–93. 10.18632/oncotarget.4494 26317899 PMC4694916

[18] Sabatier R, Finetti P, Mamessier E, Adelaide J, Chaffanet M, Ali HR, et al Prognostic and predictive value of PDL1 expression in breast cancer. Oncotarget. 2015; 6:5449–64. 10.18632/oncotarget.3216 25669979 PMC4467160

[19] Goldmann T, Kugler C, Reinmuth N, Vollmer E, Reck M. PD-L1 copy number gain in nonsmall-cell lung cancer defines a new subset of patients for anti PD-L1 therapy. Ann Oncol. 2016; 27:206–7. 10.1093/annonc/mdv510 26487587

[20] Ikeda S, Okamoto T, Okano S, Umemoto Y, Tagawa T, Morodomi Y, et al PD-L1 Is Upregulated by Simultaneous Amplification of the PD-L1 and JAK2 Genes in Non-Small Cell Lung Cancer. J Thorac Oncol. 2016; 11:62–71. 10.1016/j.jtho.2015.09.010 26762740

[21] Georgiou K, Chen L, Berglund M, Ren W, de Miranda NF, Lisboa S, et al Genetic basis of PD-L1 overexpression in diffuse large B-cell lymphomas. Blood. 2016; 127:3026–34. 10.1182/blood-2015-12-686550 27030389

[22] Twa DD, Chan FC, Ben-Neriah S, Woolcock BW, Mottok A, Tan KL, et al Genomic rearrangements involving programmed death ligands are recurrent in primary mediastinal large B-cell lymphoma. Blood. 2014; 123:2062–5. 10.1182/blood-2013-10-535443 24497532

[23] Steven H, Swerdlow EC, Harris NL, et al, editors. WHO Classification of Tumours of Haematopoietic and Lymphoid Tissues, 4th Ed Lyon, France: International Agency for Research on Cancer; 2008.

[24] Herbst RS, Soria JC, Kowanetz M, Fine GD, Hamid O, Gordon MS, et al Predictive correlates of response to the anti-PD-L1 antibody MPDL3280A in cancer patients. Nature. 2014; 515:563–7. 10.1038/nature14011 25428504 PMC4836193

[25] Azzaoui I, Uhel F, Rossille D, Pangault C, Dulong J, Le Priol J, et al T-cell defect in diffuse large B-cell lymphomas involves expansion of myeloid-derived suppressor cells. Blood. 2016; 128:1081–92. 10.1182/blood-2015-08-662783 27338100

[26] Keane C, Vari F, Hertzberg M, Cao KA, Green MR, Han E, et al Ratios of T-cell immune effectors and checkpoint molecules as prognostic biomarkers in diffuse large B-cell lymphoma: a population-based study. Lancet Haematol. 2015; 2:e445–55. 10.1016/S2352-3026(15)00150-7 26686046

[27] Gatalica Z, Bilalovic N, Palazzo JP, Bender RP, Swensen J, Millis SZ, et al Disseminated histiocytoses biomarkers beyond BRAFV600E: Frequent expression of PD-L1. Oncotarget. 2015; 6:19819–25. 10.18632/oncotarget.4378 26110571 PMC4637323

[28] Mahoney KM, Sun H, Liao X, Hua P, Callea M, Greenfield EA, et al PD-L1 Antibodies to Its Cytoplasmic Domain Most Clearly Delineate Cell Membranes in Immunohistochemical Staining of Tumor Cells. Cancer Immunol Res. 2015; 3:1308–15. 10.1158/2326-6066.CIR-15-0116 26546452 PMC4743889

[29] Joseph RW, Millis SZ, Carballido EM, Bryant D, Gatalica Z, Reddy S, et al PD-1 and PD-L1 Expression in Renal Cell Carcinoma with Sarcomatoid Differentiation. Cancer Immunol Res. 2015; 3:1303–7. 10.1158/2326-6066.CIR-15-0150 26307625 PMC4674298

